# Effect of smoking on vitamin A, vitamin E, and other trace elements in patients with cardiovascular disease in Bangladesh: a cross-sectional study

**DOI:** 10.1186/1475-2891-3-18

**Published:** 2004-10-05

**Authors:** Sam K Bashar, Amal K Mitra

**Affiliations:** 1 North South University, Dhaka, Bangladesh; 2 Department of Community Health Sciences, The University of Southern Mississippi, Hattiesburg, MS, USA

## Abstract

**Background:**

Data regarding the impact of cigarette smoking on trace elements are scarce and inconsistent. In this study, we evaluated the effect of smoking on serum concentrations of trace elements among adult males with heart disease.

**Methods:**

This cross-sectional study included 100 adults hospitalized with heart disease in Bangladesh. The major variables of interest included mean serum concentrations of trace elements and proportion of subjects with bacterial growth on throat swab culture.

**Results:**

Smokers had significantly lower serum concentrations of retinol, alpha-tocopherol, selenium, and zinc and increased concentrations of copper. Throat swab cultures were more often positive for *Streptococcus *β-*hemolyticus *in smokers than controls.

**Conclusions:**

Smoking decreases serum concentrations of trace elements. Smoking control programs are needed in Bangladesh to improve health and nutrition of the people who are already nutritionally deficient.

## Introduction

Smoking is a widely accepted practice in Bangladeshi men and is associated with socialising, sharing, and male identity [1]. According to an earlier cross-sectional study, approximately 50% of males and 3% of females are tobacco smokers in Bangladesh [2]. Although smoking is a recognized risk factor for several diseases including emphysema, chronic bronchitis, cardiovascular diseases, and cancer [3-5], very little is known about the nutritional consequences of smoking. In animal models, administration of benzo(a)pyrene, a constituent present in cigarette smoke induced vitamin A depletion [6]. Vitamin A deficiency per se causes emphysema. Some other trace elements, such as iron, zinc, and vitamin E were found to be deficient among healthy smokers compared to non-smokers. However, the available data are inconsistent regarding the effect of smoking on trace elements. In this study, we documented the effect of different doses of smoking on trace elements among hospitalized patients with heart disease in Bangladesh.

## Methods and Materials

A cross-sectional study was conducted among 100 male patients admitted to the National Institute of Cardiovascular Disease (NICVD), Dhaka, Bangladesh from January through December 1998, after obtaining informed consent from the participants. The study protocol was reviewed and approved by the Human Subjects Ethical Committee of the NICVD. The patients who had a history of smoking 10 or more cigarettes per day were considered smokers, and those who never smoked were controls. All the patients, including controls, were admitted with heart disease. The study did not include females in this study because smoking is not a norm among females in that society. All the smokers and the first 20 of the non-smokers who met the selection criteria were eligible for the study. This study included only heavy smokers who smoked at least 10 sticks per day, and excluded mild or casual smokers to leave a buffer zone of comparison between smokers and non-smokers. Patients were stratified according to their smoking status as follows: Control (*n *= 20), non-smokers; Grade I (*n *= 20), 10 – 15 sticks/day; Grade II (*n *= 30), 16 – 20 sticks/day; Grade III (*n *= 30), 21 and more sticks/day.

Body weight and height were measured at admission. Weight (kg) was measured to the nearest 0.1 kg, with participants wearing light clothing and no shoes and using a beam balance with non-detachable weights. Height (cm) was measured with a stadiometer to the nearest 0.5 cm. Body mass index (BMI) was calculated using the following formula: weight (kg) / height (m)^2^. Five ml of venous blood was obtained from each patient at admission in a test-tube, which was wrapped with aluminum foil to avoid degradation of vitamin A in light. Serum samples were separated by centrifugation, and kept stored at -20°C until further analysis. For the analyses of trace elements, serum samples were stored at separate ion-free vials. Throat swab cultures were detected for the growth of bacteria.

### Laboratory Methods

Serum retinol (vitamin A) and serum α-tocopherol (vitamin E) concentrations were determined by high performance liquid chromatography (HPLC) according to Bieri et al. [7]. In brief, serum retinol and α-tocopherol were extracted with hexane after deproteinization with absolute ethanol containing retinyl acetate and α-tocopherol acetate (Sigma Chemical Co., St Louis, MO, USA) as internal standards for retinol and α-tocopherol respectively. Retinol and α-tocopherol were separated by HPLC (model PU 4010; Pye-Unicam) on a reverse-phase C_18 _column using methanol-water (97.5:2.5, v/v) as the mobile phase. Coefficient of variation (CV) values of ten replicates from a pooled serum sample for retinol and α-tocopherol were 2.3 and 3.3% respectively.

Serum zinc was measured by flame atomic absorption spectrophotometry (AAS, Analyst 800, Perkin-Elmer, Norwalk, CT, USA) using a modification of the method described by Kirgbright [8]. Serial replication of aliquots from a pooled serum sample and quality control sera were used to check the precision and accuracy of the analytical methods. The within-run CV for zinc in a pooled serum sample was between 2.2 and 4%, based on six to seven samples in each of the five runs. Serum concentrations of copper and selenium were measured by the AAS method mentioned above.

### Statistical Methods

Data were analyzed using SPSS for windows, version 11.0 (SPSS Inc., Chicago, IL). Descriptive statistics of the major variables of interest were calculated to determine the distribution of the data. All the variables except serum iron concentrations were normally distributed. Variables between smokers and non-smokers were compared by Student t-test for continuous data and by Chi-square test for categorical data. A probability level of 5% was considered statistically significant.

## Results

Of the 110 patients enrolled, 10 dropped out; eight had incomplete data, one withdrew early, and one left the hospital without notice. The mean ± SD age of the study patients was 43.67 ± 6.79 y (range, 28 – 57 y). The groups did not differ significantly in terms of age and anthropometric measurements (Table 1).

**Table 1:** Baseline characteristics of the study subject

	Control (Non-smoker) *n *= 20	Smoker			All *n *= 100
			
		Grade I 10–15 sticks/d *n *= 20	Grade II 16–20 sticks/d *n *= 30	Grade III 21+ sticks/d *n *= 30	
Age (year)	41.60 ± 8.30	44.40 ± 7.52	43.57 ± 5.85	44.67 ± 6.06	43.67 ± 6.79
Weight (kg)	60.95 ± 3.32	63.25 ± 3.40	62.50 ± 3.64	62.90 ± 2.81	62.46 ± 3.34
Height (m)	1.64 ± 0.03	1.65 ± 0.03	1.65 ± 0.03	1.63 ± 0.04	1.65 ± 0.03
BMI	22.32 ± 1.35	23.11 ± 1.31	22.90 ± 1.63	23.17 ± 1.10	22.91 ± 1.38

Values are mean ± *SD*.

Serum retinol concentrations were below normal (0.70 μmol/L) among all smokers and the majority (60%) of the controls. Table 2 shows that the smokers who smoke 16 sticks or more cigarettes per day had significantly lower concentrations of serum retinol compared with controls. Percentage decrease of alpha-tocopherol was most striking of all the trace elements. Zinc concentrations did not change among grade I and grade II smokers but decreased among grade III smokers compared with controls. Concentrations of copper increased but selenium decreased among smokers than controls.

**Table 2:** Effect of smoking on serum concentrations of retinol, alpha-tocopherol and other trace elements

Trace element (μmol/L)	Control (Non-smoker) *n *= 20	Smoker		
		
		Grade I 10–15 sticks/d *n *= 20	Grade II 16–20 sticks/d *n *= 30	Grade III 21+ sticks/d *n *= 30
Retinol	.72 ± .06	.66 ± .12	.45 ± .09*	.28 ± .07*
Alpha-tocopherol	13.5 ± 1.6	10.0 ± .7**	8.6 ± 1.9***	7.9 ± 1.2***
Copper	.44 ± .25	.46 ± .16	.61 ± .19*	.62 ± .27*
Selenium	.013 ± .001	.004 ± .001*	.003 ± .001*	.003 ± .001*
Zinc	.46 ± .23	.46 ± .15	.46 ± .20	.42 ± .15*

Values are mean ± *SD*. *P < .05; **P < .01; ***P < .001 compared with the control.

A significantly higher proportion of smokers compared with controls had bacterial growth on their throat cultures, mostly due to *Streptococcus *β-*hemolyticus *(Table 3).

**Table 3:** Effect of smoking on bacterial growth on throat swab culture

Organism	Control (Non-smoker) *n *= 20	Smoker		
		
		Grade I 10–15 sticks/d *n *= 20	Grade II 16–20 sticks/d *n *= 30	Grade III 21+ sticks/d *n *= 30
*Streptococcus *β *hemolytica*	2.8	70.5	71.4	72.5
*Aerobacter aerogenes*	12.2	25.4	26.7	27.0
No growth	85.0	4.1	1.9	1.0

Values are percentages. *P *< .001 for all values of smokers compared with the control.

## Discussion

In this study, adult male smokers with heart disease had significantly decreased serum concentrations of retinol, alpha-tocopherol, and selenium, and increased concentrations of copper, compared to non-smokers. Depression of trace elements in blood was more with increasing doses of smoking.

In a study in Turkey, plasma selenium, copper, zinc and iron concentrations, and the activities of related erythrocyte antioxidative enzymes copper-zinc superoxide dismutase (Cu-Zn SOD), catalase, and glutathione peroxidase (GSH-Px) were measured in tobacco smokers and compared with those of nonsmokers [9]. Plasma thiocyanate levels were measured as an index of smoking status. While plasma copper concentration and erythrocyte Cu-Zn SOD activity were significantly higher, plasma selenium concentration and erythrocyte GSH-Px activities were significantly lower in tobacco smokers than in nonsmokers. We did not measure antioxidative enzyme levels in blood; however, our study had consistency with earlier findings of decreased serum concentrations of selenium and increased concentrations of copper among smokers. Kocyigit et al. [9] did not observe any significant effect of smoking on zinc or iron status. Our observation of a significantly depressed zinc status only among heavy smokers (those who smoked 21 or more sticks per day) compared to non-smokers was consistent with findings of Uz et al. in Turkey [10].

Several studies documented that smoking may increase oxidative stress and impair oxidant defense system [11]. Serum selenium glutathione peroxidase, glutathione reductase, and extracellular superoxide dismutase activities were found lower in smokers than in non-smokers. Serum ascorbic acid and folate concentrations were lower in smokers than in non-smokers, whereas serum thiobarbituric acid-reactive substances (TBARS) were higher. However, Kim et al. (2003) did not observe any effect of smoking on serum copper, iron, and magnesium concentrations [11].

In a later study, Kim et al. (2004) further evaluated the influence of short– and long-term cigarette smoking on blood antioxidant status among Korean teenage girls (aged 14 to 18 y) and adult males (aged 36 to 51 y) [12]. Extracellular superoxide dismutase activities and concentrations of serum vitamin C and folate were lower in both short-term and long-term smokers. Serum copper concentrations were higher only among long-term smokers compared to non-smokers. In our study, we observed increased serum concentrations of copper among grade II and grade III smokers (those who smoked 16 or more sticks per day) but not among grade I smokers (those who smoked 10–15 sticks per day), as compared to non-smokers. Both the studies suggest that probably an increasing dose of smoking modify serum copper status more compared to those who smoke less or do not smoke at all. However, cigarette smoking, irrespective of dose or duration, had negative effects on antioxidant status in the Korean study [12].

Increasing evidence suggests that smoking is a causal factor for coronary heart disease and stroke. In a prospective study in Japan [4], 19,782 men and 21,500 women aged 40 to 59 years who were free of prior diagnosis of stroke, coronary heart disease, or cancer and reported their smoking status were followed. During a 461,761 person-year follow-up, relative risks (95% CIs) for current smokers compared with never-smokers were 1.27 (1.05 to 1.54) for total stroke, 0.72 (0.49 to 1.07) for intraparenchymal hemorrhage, 3.60 (1.62 to 8.01) for subarachnoid hemorrhage, and 1.66 (1.25 to 2.20) for ischemic stroke.

One of the limitations of our study is that it is difficult to establish any causal association of heart disease and deficiency of trace elements or increased isolation of *Streptococcus *β-*hemolyticus *among our study subjects, because it is a cross-sectional study. However, epidemiologic evidence has suggested a modifying role for antioxidant micronutrients, including tocopherols and carotenoids, in atherosclerosis and heart disease. In an experimental study, Handelman et al. (1996) exposed freshly obtained human plasma to the gas phase of cigarette smoke to assess its effects on tocopherols, carotenoids, and retinol. Exposure to cigarette smoke led to the depletion of most of the lipophilic antioxidants in human plasma [13].

In addition to the impact on health, tobacco smoking represents a major economic burden for impoverished Bangladeshis. Average male cigarette smokers spend more than twice as much on cigarettes as per capita expenditure on clothing, housing, health and education combined. The typical poor smoker could easily add over 500 calories to the diet of one or two children with the daily tobacco expenditure [14]. It may be noted that most of the study subjects were undernourished, as indicated by an average BMI of 23. Strong tobacco control measures are needed in the context of Bangladesh to decrease tobacco expenditures and thus significantly increase resources and improve health and nutrition of the people.

## Conclusion

This study demonstrated that increasing amount of cigarette smoking negatively impact serum concentrations of retinol, alpha-tocopherol, selenium, and zinc. Cigarette smoking may act as an important adjunct to the deficiency of those trace elements in a population who are already nutritionally compromised.

## Competing interests

The authors declare that they have no competing interests.

## Authors' contributions

SKB participated in the design of the study and collected the samples. AKM performed the statistical analysis and drafted the manuscript.
